# Evaluation of the Chemical Composition and Antioxidant Activity of Mulberry (*Morus alba* L.) Fruits from Different Varieties in China

**DOI:** 10.3390/molecules27092688

**Published:** 2022-04-21

**Authors:** Tao Chen, Fei-Fan Shuang, Qing-Yue Fu, Yu-Xiong Ju, Chen-Man Zong, Wei-Guo Zhao, Dong-Yang Zhang, Xiao-Hui Yao, Fu-Liang Cao

**Affiliations:** 1 College of Biotechnology and Sericultural Research Institute, Jiangsu University of Science and Technology, Zhenjiang 212100, China; zjchentao@sina.com (T.C.); 18852895549@163.com (F.-F.S.); 18860106781@163.com (Q.-Y.F.); just202310032@163.com (Y.-X.J.); 18852856090@163.com (C.-M.Z.); zhaoweiguo1506@163.com (W.-G.Z.); zhangdongyang1987@126.com (D.-Y.Z.); 2 Co-Innovation Centre for Sustainable Forestry in Southern China, College of Forestry, Nanjing Forestry University, Nanjing 210037, China

**Keywords:** mulberry fruit, varieties and regions, anthocyanin, chemical composition, antioxidant activity

## Abstract

Mulberry (*Morus alba* L.) fruit is a fruit with nutritional and medicinal value. It is widely cultivated in different regions of China, which may result in differences in its chemical composition. In this research, 25 mulberry fruit samples from six provinces in China were investigated. The contents of anthocyanins were evaluated by high-performance liquid chromatography (HPLC). The contents of two main anthocyanins, cyanidin-3-*O*-glucoside (C3G) and cyanidin-3-*O*-rutinoside (C3R), ranged from 0.656 ± 0.006 mg/g to 4.367 ± 0.243 mg/g and from 0.226 ± 0.007 mg/g to 1.649 ± 0.013 mg/g, respectively. Additionally, the contents of total phenolic, total flavonoid, vitamin C, titratable acids, reducing sugars and antioxidant capacity (FRAP, DPPH, scavenging and hydroxyl radical scavenging activity) were also assessed. The results and principal component analysis showed that the Zhongsang 5801 variety from Sichuan, Dechang had the greatest health value with the highest active compound contents. Based on our analysis, the variety from Sichuan, Dechang is a high-quality plant source for mulberry fruit cultivation. This research provides a basis for the rational development and utilization of mulberry fruit resources in China.

## 1. Introduction

In recent decades, one of the most popular trends in food-based health products and medical research is the use of natural bioactive substances from plants as a source of anti-oxidant, anti-inflammatory, anti-cancer, anti-diabetic, and weight-loss chemicals [1,2,3,4]. Mulberry (*Morus alba* L.), belonging to the genus Morus of the family Moraceae, is widely distributed in Asia, Europe, North America, South America, and Africa. In China, mulberry trees have been planted for sericulture for 5000 years, and are widely distributed in many provinces. The fruits of the mulberry tree can not only be eaten fresh or made into manufactured food but also can be used as a raw material for some health products. As early as 2012, mulberry fruit was included in the national homologous catalogue of medicines and foods [5].

Traditional Chinese medicine books have recorded the health effects of mulberry [6], with the main functions of the mulberry fruit cited as tonifying the liver and kidneys, nourishing the yin and blood, and improving eyesight. Modern medical research has shown that mulberry fruit can supplement nutrition, strengthen the spleen and stomach to help digestion, enhance immunity, and prevent vascular sclerosis [7]. Mulberry fruit contains stilbenoids (e.g., resveratrol), flavonoids (e.g., rutin, quercetin and kaempferol), phenolic acids (e.g., protocatechuic acid, caffeoylquinic acids), and anthocyanins (e.g., cyanidin and pelargonidin glycosides) that function as natural antioxidants in the human body [8,9,10,11,12,13]. Anthocyanins are widely distributed plant pigments that are present in the cytosol and gives flowers, fruits, stems, leaves, and root organs different colors. As a natural pigment, anthocyanin is safe and non-toxic with many health care functions in the human body. In addition, anthocyanins inhibit the growth of food-borne pathogens [14], and play an important role in scavenging free radicals in vivo, conveying protections that are anti-tumor, anti-cancer, anti-inflammatory, promoting weight loss and vision health, and preventing chronic diseases and diabetes [15]. Previous studies [16,17,18] have reported that the main anthocyanins in mulberry fruit are cyanidin-3-*O*-glucoside (C3G), cyanidin-3-*O*-rutinoside (C3R), pelargonidin-3-*O*-glucoside (P3G), and pelargonidin-3-*O*-rutinoside (P3R), and the contents of C3G and C3R were found to account for more than 98% of the total anthocyanins. In order to accurately measure the contents of different anthocyanins in mulberry fruit, an appropriate separation is crucial [19]. In this study, we used an HPLC method to detect and quantify the major anthocyanins in mulberry fruit.

Mulberry fruit is cultivated widely in China. Long-term and large-scale planting has been carried out in many provinces, such as Jiangsu, Zhejiang, Sichuan, Chongqing, Liaoning, and Heilongjiang. However, these mulberry producing areas have obvious differences in climate, terrain and altitude, which can impact the chemical composition and nutritional value of plants [20,21]. Meanwhile, some studies reported that the nutrients and phytochemicals in mulberries are not only related to the species or cultivars, but also with the region of cultivation [22,23]. Most previous studies focused on the study of the component differences of mulberry fruit in a few regions and varieties, but there are few relevant studies on the component differences of mulberry fruit in the main growing regions of China [22,24,25]. This research focused on the analysis and comparison of the physico-chemical and antioxidant properties of different cultivars from eight regions in China, hoping to provide valuable information for the rational utilization of mulberry cultivars and the development of related health products. The physico-chemical and antioxidant properties measured were also used as descriptors for principal component analysis (PCA) in order to differentiate the analyzed mulberry cultivars.

## 2. Results and Discussion

In this study, different varieties of mulberry fruits from Jiangsu, Zhejiang, Sichuan, Chongqing, Liaoning, and Heilongjiang provinces were investigated. In order to evaluate the quality and taste of these mulberry fruits, the contents of total phenolic, total flavonoid, main anthocyanin, vitamin C, titratable acidity and reducing sugar were measured. Moreover, an HPLC method was used to analyze the contents of four main anthocyanins (cyanidin-3-*O*-glucoside (C3G), cyanidin-3-*O*-rutinoside (C3R), pelargonidin-3-*O*-glucoside (P3G), and pelargonidin-3-*O*-rutinoside (P3R)). The antioxidant activity of the extract was measured using a total antioxidant capacity assay kit (FRAP), 1,1-diphenyl-2-picrylhydrazyl (DPPH) radical scavenging, and hydroxyl radical scavenging rate tests. To better explain the relationship among bioactive components, varieties and regions, we used principal component analysis (PCA) to analyze the correlations. The following sections are specifically discussed.

### 2.1. Total Phenolic and Flavonoid Contents in Mulberry

In this study, total phenolic (TPC) and total flavonoid (TFC) contents were determined. An abbreviated list of variety names of mulberry fruits is provided in Table 1.

The TPC ranged from 0.727 ± 0.101 mg GAE/g (Shuangcheng, QY) to 6.307 ± 0.541 mg GAE/g (Dechang, ZS) (Table 2). The TPC of six different cultivars, namely ML, HY, ZS, YS, HG, and DS from Dechang and Chongqing were above 4 mg GAE/g. The samples from Dechang and Chongqing had higher TPC values than other regions. Contessa et al. [26] reported TPCs in strawberry (3.9867 mg GAE/g) and berry fruits (1.9698–3.6215 mg GAE/g) that was much lower than the mulberry fruit TPC found in this study.

The TFC varied from 0.199 ± 0.023 mg RE/g to 3.662 ± 0.269 mg RE/g. QY planted in Shuangcheng had the lowest value, and ZS planted in Dechang had the highest value. TPC and TFC had a similar geographic trend (Table 2).

Dechang has a subtropical plateau monsoon climate with an average annual temperature of 17.7 °C, an average annual precipitation of 1049 mm, a frost-free period of more than 300 days, and 2147 h of annual sunshine. This environment is conducive to the accumulation of active ingredients in mulberry fruit [23]. This could explain why ZS from Dechang has higher TPC and TFC values than the others.

### 2.2. The Vitamin C, Reducing Sugar, and Titratable Acidity Contents

The vitamin C content varied from 3.310 ± 0.736 mg/100 g to 45.712 ± 2.775 mg/100 g, in which TS from Dechang had the highest vitamin C content and HG from Chongqing had the lowest (Table 2).

Sugar and organic acids have an important effect on fruit flavor qualities. We found a large difference in the sugar and organic acid contents among different varieties. The titratable acidity varied from 0.397 ± 0.023% (Dechang, JL) to 7.479 ± 0.495% (Chongqing, Zijin6). The titratable acidity of four different cultivars including HY planted in Dechang and YS, AY, and ZJ from Chongqing was more than 5%. Imran et al. [27] found that the titratable acidity in mulberry fruits from Pakistan ranged from 0.84% to 2%. Calín-Sánchez et al. [28] reported that titratable acidity in mulberry ranged from 0.93% to 2.65% in Spain. The titratable acidity detected in our study was much higher than that detected in these previous studies.

Previous research has demonstrated that environmental conditions can influence sugar synthesis [29]. In this study, the reducing sugar content varied from 0.515 ± 0.027% (Anji, DS) to 16.961 ± 0.037% (Dechang, 831A) (Table 2). The 831A planted in Dechang was the only cultivar with a reducing sugar content above 10%. In contrast, the reducing sugar content values of the HG from Fengcheng and the DS from Anji were both lower than 1%.

### 2.3. Determination of Main Anthocyanin Contents by HPLC

**Figure 1:**
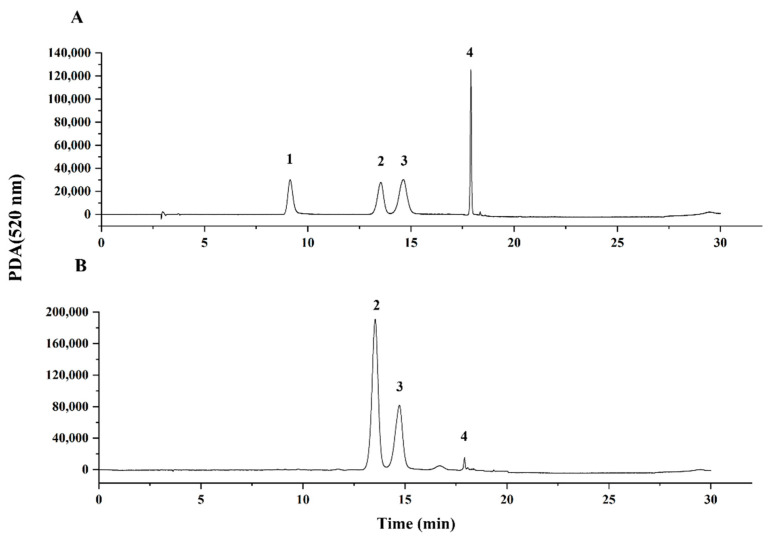
HPLC chromatograms of (**A**) the four standard substances of anthocyanins and (**B**) mulberry fruit extracts (Dechang, ZS) were recorded at 520 nm using a PDA detector. Peak 1, pelargonidin-3-*O*-glucoside (P3G); 2, cyanidin-3-*O*-glucoside (C3G); 3, cyanidin-3-*O*-rutinoside (C3R); and 4, pelargonidin-3-*O*-rutinoside (P3R).

An HPLC method was established to detect the four main anthocyanins (C3G, C3R, P3G, and P3R) in mulberry fruit. Figure 1A shows the HPLC chromatograms of the four anthocyanin mixture standards, and retention times for P3G, C3G, C3R, and P3R were 9.71 min, 13.96 min, 15.56 min, and 18.02 min, respectively. In this study, not all mulberry fruits contained these four kinds of anthocyanin (Figure 1B). The contents of C3G and C3R were measured in almost all the samples, but the content of P3R was hardly detected in most samples, and the content of P3G was below the detection limits for all the mulberry samples. A similar result was also found by other studies [16].

**Figure 2:**
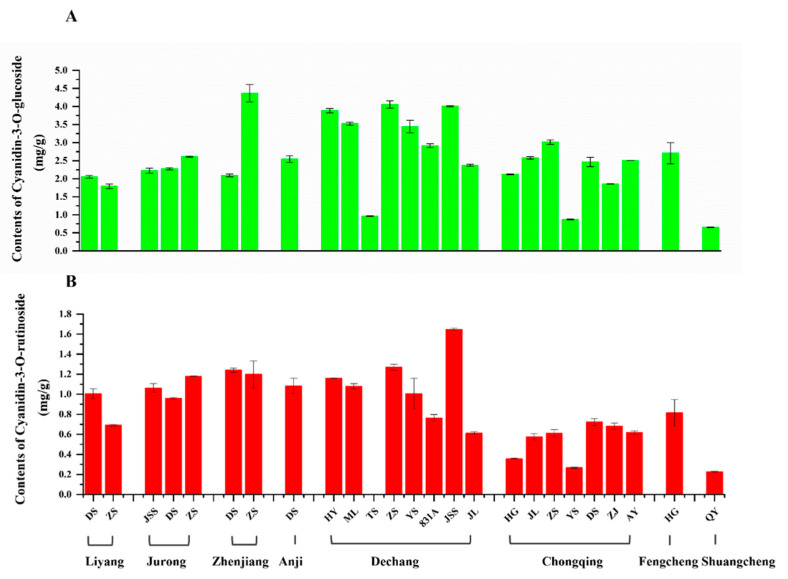
The contents of cyanidin-3-*O*-glucoside (**A**) and cyanidin-3-*O*-rutinoside (**B**) in mulberry fruits among varieties from different areas in China.

The contents of C3G, C3R, and P3G are shown in Table 3. The C3G content ranged from 0.656 ± 0.006 mg/g (Shuangcheng, QY) to 4.367 ± 0.243 mg/g (Zhenjing, ZS). Samples from Dechang showed a higher content of C3G than other places except TS. For C3R, TS from Dechang was below the detection limits. The C3R content had a similar geographic trend to C3G and ranged from 0.226 ± 0.007 mg/g (Shuangcheng, QY) to 1.649 ± 0.013 mg/g (Dechang, JSS). The contents of C3G and C3R had a consistent trend as well (Figure 2). P3R was detected in ZS, YS, 831A, and JL from Dechang, and ranged from 0.319 ± 0.014 mg/g to 0.432 ± 0.038 mg/g (Table 3). The P3R content of YS was the highest and JL was the lowest. These results showed that the anthocyanin contents of mulberry fruit in Dechang were higher than those in other regions. Kamiloglu et al. [17] also reported that four kinds of anthocyanins, namely C3G, C3R, P3G, and P3R existed in mulberry fruit, and the contents recorded were similar to our results.

In this research, the main anthocyanin content was seen as the sum of three kinds of anthocyanin which was detected by HPLC. The MAC ranged from 0.882 ± 0.006 mg/g to 5.737 ± 0.052 mg/g (Table 3), and this agreed with a previous study which reported that the TAC ranged from 0.51 to 28.61 mg/g [30]. QY planted in Shuangcheng had the lowest value. ZS planted in Dechang had the highest value, and was 6.5 times higher than that of QY.

### 2.4. Antioxidant Capacity

**Figure 3:**
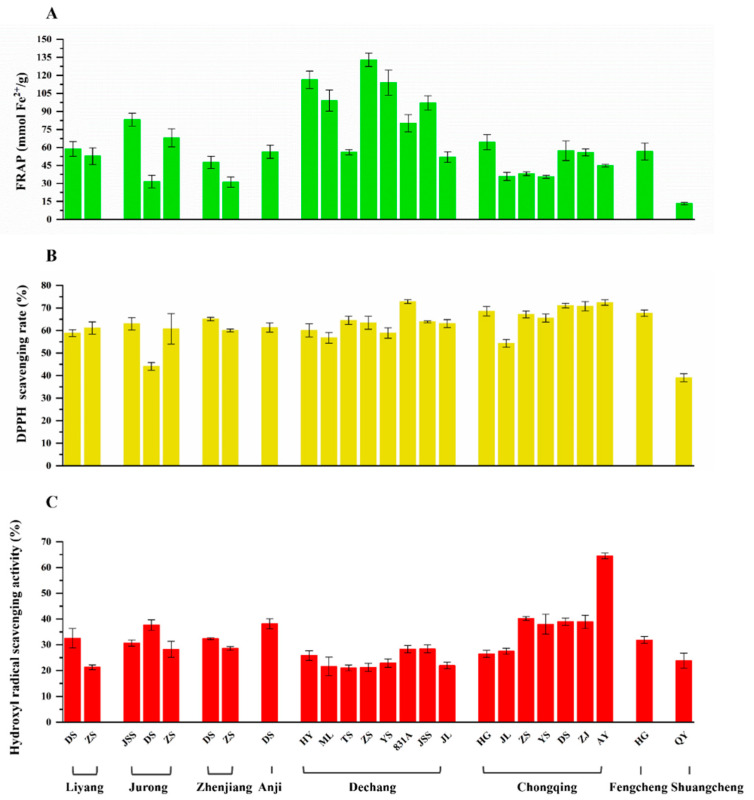
The antioxidant activity: FRAP (**A**), DPPH radical-scavenging activity (**B**), hydroxyl radical-scavenging activity (**C**) in mulberry fruits among varieties from different areas in China.

In the human body, almost 2–3% of oxygen is converted into reactive oxygen species (ROS) and free radicals, which can trigger enhanced oxidative damage to various biomolecules, such as DNA, proteins, small cellular molecules, and membrane lipids [31]. Anthocyanins, phenolic compounds, and other flavonoid compounds in mulberry are linked with oxidative damage prevention. For the purpose of comparison, the in vitro antioxidant activity was detected using three methods (FRAP, DPPH, and hydroxyl radicals scavenging activity assays) (Figure 3).

For the FRAP assays that tested the total antioxidant capacity, the results were expressed using the concentration of a FeSO_4_ standard solution (mmol Fe^2+^/g). The FRAP values ranged from 13.321 ± 1.014 mmol Fe^2+^/g (Shuangcheng, QY) to 133.083 ± 5.603 mmol Fe^2+^/g (Dechang, ZS), with the highest value being 10 times higher than the lowest one. According to the data, the total antioxidant activity had obvious regional difference. The samples from Dechang had the best total antioxidant capacity (by FRAP), followed by those from Jurong, Fengcheng, Anji, Liyang, Chongqing, Zhenjiang, and Shuangcheng (Figure 3).

For the DPPH assay, in order to measure the antioxidant capacity of different samples, mulberry fruit extracts of the same concentration (5 mg/mL) were used and compared via the DPPH free radical scavenging rate. The results varied from 39.077 ± 1.758% (Shuangcheng, QY) to 72.846 ± 0.796% (Dechang, 831A), and the highest DPPH free radical scavenging rate was 1.9 times higher than the lowest one. As can be seen in Figure 3, the DPPH free radical scavenging rate of the samples from Jurong, DS was 44.118 ± 1.722%, and that of the samples from Shuangcheng, QY was 39.077 ± 1.758%, with these being relatively lower than those obtained for other samples in the experiment. In a previous study, Korean mulberry extract scavenged 60% DPPH radicals at 200–683 μg depending on the different cultivars, showing a similar antioxidant effect to our mulberry fruits [21].

The hydroxyl radical scavenging activity assay is a widely used method to determine antioxidant activity. The scavenging rate of hydroxyl radicals ranged from 21.002 ± 1.156% (Dechang, TS) to 64.488 ± 1.074% (Chongqing, AY), and the highest hydroxyl radicals scavenging activity was 3.1 times higher than the lowest one. As seen in Figure 3C, we found that samples from Chongqing had better hydroxyl radical scavenging activity than other regions.

### 2.5. Principal Component Analysis

Principal component analysis (PCA) was used to study the relationship between antioxidant activity and active ingredients. The parameters analyzed included the contents of total phenolic (TPC), total flavonoid (TFC), main anthocyanin (MAC), vitamin C (VC), reducing sugar (RS), titratable acidity (TA), FRAP, DPPH, the hydroxyl radical scavenging rate (OH scavenging), cyanidin-3-*O*-glucoside (C3G), and cyanidin-3-*O*-rutinoside (C3R). Two principal components were obtained that explained 57.87% of the whole data variance: PC 1 and PC2 accounted for 38.07% and 19.80% of the total variance, respectively. Sánchez-Salcedo et al. [32] obtained 52.28% of explained variance which is similar to our study. The correlation coefficients of the variables are displayed in Table 4.

PC 1 = 0.636 TPC + 0.770 TFC + 0.899 MAC + 0.129 VC + 0.370 RS − 0.124 TA + 0.890 FRAP + 0.229 DPPH − 0.327 OH scavenging + 0.870 C3G + 0.712 C3R(1)

PC 2 = 0.526 TPC + 0.101 TFC − 0.101 MAC + 0.202 VC − 0.389 RS + 0.874 TA + 0.124 FRAP + 0.704 DPPH + 0.613 OH scavenging − 0.029 C3G − 0.197 C3R(2)

**Figure 4:**
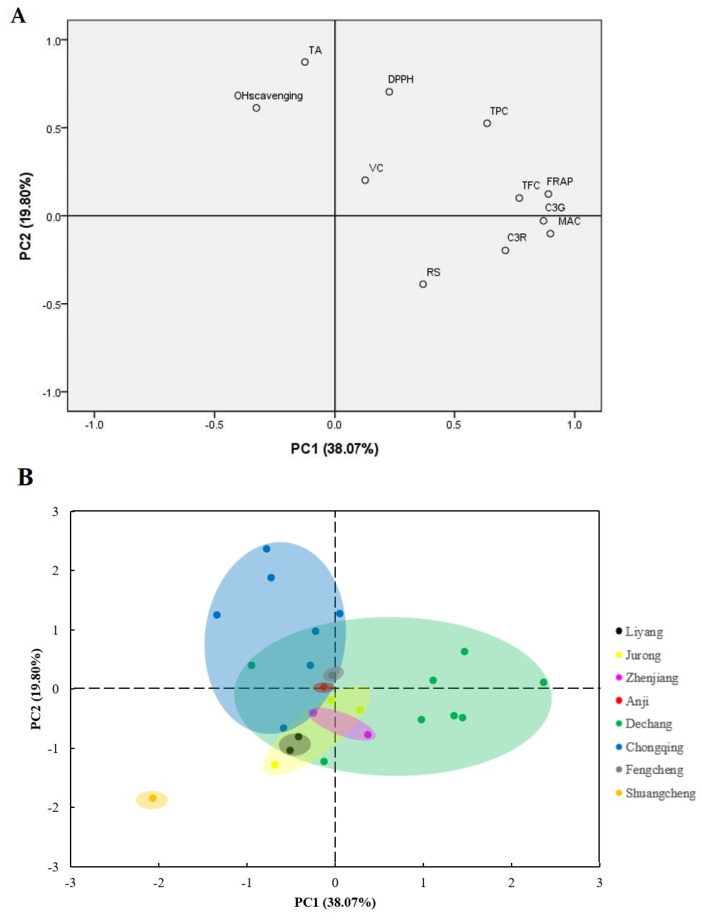
Principal component analysis loading plot of TPC, TFC, antioxidant activity, and active compounds from different locations in China (**A**), and the principal component analysis score plot of different mulberry samples in China (**B**).

Based on the equations above, the first principal component (PC1) correlated well with the MAC, FRAP, and C3G with loadings of 0.899, 0.890, and 0.870 obtained, respectively. PC2 seemed to be related to TA and DPPH with loadings of 0.874 and 0.704 obtained, respectively. As can be seen in Figure 4A, FRAP, TPC, TFC, MAC, C3G and C3R was closely related to PC1, indicating that PC1 had a good correlation with the antioxidant activity and components. The results also suggested that TPC, TFC, MAC, C3G and C3R were the main contributors to FRAP (Figure 4A and Table 4). PC2 was strongly related to TA, DPPH and OH radical scavenging, and it was poorly correlated with C3G. A strong correlation between TPC and DPPH suggested that TPC played a major role in DPPH free radical scavenging. There was a significant correlation between TFC and OH radical scavenging ability, indicating that TFC played a major role in OH radical scavenging.

Figure 4B shows that the distribution of mulberry fruits from the same region was relatively close, and the scores in PC1 and PC2 were similar, while the scores of mulberry fruits from different regions were different, thus showing a regional difference. Therefore, the region has a great influence on the content of active components of mulberry fruit, and this then affects its antioxidant capacity. Further, by overlapping the plots A and B from Figure 4, it is evident that most of the samples from Dechang had strong correlation with FRAP, TPC, TFC, MAC, C3G and C3R, indicating that the samples in this area have a high content of active components and good antioxidant activity. The samples from Chongqing were well correlated with DPPH and OH radical scavenging, revealing that the samples in this area had strong free radical scavenging activity. From these results, the mulberry fruit of Dechang had more active components and a higher FRAP antioxidant capacity, which may be an excellent source of health-promoting components in health products. At the same time, Chongqing mulberry had an excellent free radical scavenging ability.

## 3. Materials and Methods

### 3.1. Chemicals and Reagents

Standards of C3G, C3R, P3G, P3R, gallic acid, and rutin were purchased from Spring and Autumn Co. (Nanjing, China). DPPH (2,2-diphenyl-1-picrylhydrazyl) and formic acid (HPLC grade, 98%) were obtained from Aladdin (Shanghai, China). The purity of all standard products was ≥98%. The Folin-Ciocalteu reagent was from Sigma Aldrich (St. Louise, MO, USA). Acetonitrile (HPLC grade, 98%) was obtained from Tedia Co. (Fairfield, OH, USA). Total antioxidant capacity assay kits (FRAP method) were purchased from the Beyotime Institute of Biotechnology (Shanghai, China). Reducing sugar assay kits were obtained from the Leagene Biotechnology Co., Ltd. (Beijing, China). Water was prepared using a Milli-Q purification system from Millipore (Bedford, MA, USA).

### 3.2. Plant Material

**Figure 5:**
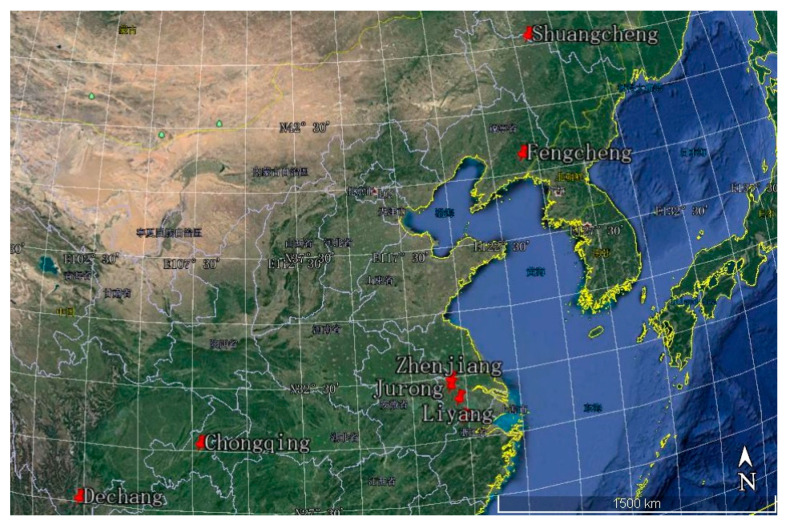
Different collection area of mulberry fruit (*Morus alba* L.) in China. Chinese means province in China.

The samples from eight areas (Liyang, Jurong, Zhenjiang, Anji, Dechang, Chongqing, Fengcheng, and Shuangcheng) in six provinces (Jiangsu, Zhejiang, Sichuan, Chongqing, Liaoning, and Heilongjiang) were collected during the mature period, from April to June 2017. The 25 samples collected were identified by Professor Weiguo Zhao from the College of Biotechnology and Sericultural Research Institute, Jiangsu University of Science and Technology, Zhenjiang, Jiangsu, China. The eight collection areas are labeled in Figure 5, which was drawn using Google Earth software (Google Inc., Mountain View, CA, USA).

### 3.3. Extraction of the Mulberry Fruit

Each fresh mulberry fruit (3.0 g) was mixed with 70% aqueous ethanol (30 mL) and ground to obtain the mixture. The mixture was sonicated for 30 min at room temperature in ultrasonic extractors (KQ-2200DB, 100 W, Kunshan, China) and then centrifuged at 8000 r/min for 15 min to collect the supernatant. The bottom mulberry fruit residue was extracted twice according to the above method. The three extracts were collected, transferred to a 100-mL volumetric flask and diluted to volume with 70% ethanol. 

### 3.4. Total Phenolic Content

The Folin-Ciocalteu method [33] was used to determine the total phenolic content (TPC) of the mulberry fruits with a slight modification. Briefly, the Folin-Ciocalteu reagent (2N) was diluted 20 times with deionized water to create the Folin-Ciocalteu working solution. Then, 1.8 mL of Folin-Ciocalteu working solution was added to 40 μL of the samples and the blank (40 μL 70% aqueous ethanol). This mixture was allowed to react for 5 min at room temperature. Afterward, 1.2 mL of 7.5% sodium carbonate solution was added into the mixture and allowed to react at room temperature for 1 h in the dark. Finally, the absorbance was measured at 765 nm. The standard substance was gallic acid (y = 0.6812x + 0.0314, R^2^ = 0.9975), and the experimental total phenolic contents were expressed as gallic acid equivalents (mg GAE/g).

### 3.5. Total Flavonoid Content

The total flavonoid content (TFC) was determined by an aluminum chloride method [34] using rutin as a reference substance with a slight modification. Briefly, 1 mL extracts and blank (the solvent without samples dissolved) were mixed with 3 mL of 0.1 mol/L CH_3_COOK and 2 mL of a 0.1 mol/L AlCl_3_ solution. Afterward, the mixture was allowed to react for 20 min and then diluted with 70% aqueous ethanol (*v*/*v*) to a final volume of 10 mL. Finally, the absorbance was measured at 510 nm, and the results were expressed as rutin equivalents (mg RE/g); the standard curve was y = 1.4715x + 0.0364 (R^2^ = 0.9998).

### 3.6. Anthocyanin Content

#### 3.6.1. Extraction of Mulberry Anthocyanins

Each kind of the fresh mulberry fruit was accurately weighed as 3.0 g and mixed with 30 mL 70% aqueous ethanol acidified with 1% hydrochloric acid (*v*/*v*), and then ground to obtain the mixture. The mixture solution was sonicated for 30 min in ultrasonic extractors and then centrifuged at room temperature at 8000 r/min for 15 min. This extraction process of the same sample was repeated twice. The three extracts were collected and the volume brought to 100 mL with 70% aqueous ethanol acidified with 1% hydrochloric acid (*v*/*v*).

#### 3.6.2. HPLC Analysis

The mulberry fruits were extracted with ethanol acidified with hydrochloric acid (1%) and filtered with a 0.22 μm pore size membrane filter. The four main anthocyanins were determined by HPLC-PDA (Thermo-Accela, Watertown, MA, USA). An HPLC column (Sunfire C18, 4.6 × 250 mm, 5 μm) was used. The mobile phase included acetonitrile as solution A and a 1% formic acid aqueous solution as solution B. The mobile phase gradient was as follows: 0–5 min, 5–10% A; 5–10 min, 10–12% A; 10–14 min, 12% A; 14–20 min, 12–70% A; 20–25 min, 70% A; and 25–26 min, 70–5% A. The sample injection volume was set as 1 μL; the flow rate was set to 1000 μL/min; the column temperature was 26 °C. The anthocyanin content was determined by absorbance at 520 nm. The standard curves for C3G (y = 7331.9x − 18286, R^2^ = 0.9999), C3R (y = 6922x − 19497, R^2^ = 0.9998), P3G (y = 3605.4x + 3205.2, R^2^ = 0.9981), and P3R (y = 4514.6x + 14698, R^2^ = 0.9938) were used to calculate the contents. Generally speaking, total anthocyanins content refers to the sum of all anthocyanins in a plant. In this paper, the main anthocyanin content (MAC) refers to the sum of three main anthocyanins that can be detected.

#### 3.6.3. Method Validation

The limit of detection (LOD) and the limit of quantification (LOQ) of each anthocyanin were obtained with signal-to-noise ratios. The LOD is three times the signal-to-noise ratio, and the LOQ is ten times the signal-to-noise ratio. The LODs of P3G, C3G, C3R and P3R were 17.5, 25, 17.5 and 7.5 μg/mL, respectively. The LOQs of P3G, C3G, C3R and P3R were 50, 50, 50 and 20 μg/mL, respectively.

### 3.7. Vitamin C Content

The vitamin C content in mulberry was determined directly by Chinese Standard GB 5009.86−2016 [35]. The mulberry fruit (10 g) was added into 100 mL metaphosphoric acid (2%, *w*/*v*) and homogenized to obtain the leaching solution. The leaching solution (5 mL) was combined with 5 mL of pH 4.0 sodium acetate buffer solution (the mixture of sodium acetate solution and glacial acetic acid) and 2 mL of 2,6-dichloroindophenol solution (containing 52 mg sodium bicarbonate, 50 mg 2,6-dichloroindophenol and 200 mL water). After intense shaking, 10 mL of xylene was immediately added to the mixture and shaken for 20 s. After that, 200 µL of the solution was drawn from the xylene layer and the absorbance of the solution was measured at 500 nm. The vitamin C content was calculated as follows:

(3) $$Vitamin\;C\;content\left(\mathrm{mg}/100g\right)=\frac{\left(2-V\right)\times T\times A}{W}\times 100$$

where “2” is the volume of 2,6-dichloroindophenol solution; *V* is the volume of 2,6-dichloroindophenol solution (mL) calculated on the standard curve (y = 0.0686x + 0.0395, R^2^ = 0.9946); *T* is the titration degree of 2,6-dichloroindophenol solution (mg/mL); *A* is the diluted multiples; and *W* is the weight of the sample (g). The entire experiment was able to be completed within 30 min.

### 3.8. Reducing Sugar Content

The contents of reducing sugar of the mulberry samples were detected using a reducing sugar content assay kit (Beijing Leagene Biotechnology Co., Ltd., Beijing, China). First, each mulberry fruit sample was accurately weighed as 0.5 g, ground in the mortar, and mixed with 3 mL of distilled water and then the mixture was transferred to a conical flask. Next, the mortar was rinsed with 12 mL of distilled water three times, and the eluate was transferred into the same conical flask. After that, the solution was kept in a water bath at 5070 for 30 min with full stirring, and then the mixture was centrifuged at 4000 r/min for 5 min. The distilled water (20 mL) was added to the residue, and then the mixture was shaken and centrifuged at 4000 r/min for 5 min again. The supernatant obtained by carrying out centrifugation twice was combined and this fixed the volume to 100 mL, with distilled water as the reducing sugar test solution.

The assay kit consists of a glucose standard solution (1 mg/mL) and 3,5-dinitrosalicylic acid (DNS) test solution. The DNS test solution (2 mL) was added into 1 mL of the reducing sugar test solution. After being heated in a boiling water bath for 5 min, the mixture was cooled to room temperature. Afterwards, 9 mL of distilled water was added to the mixture. Finally, the absorbance was measured at 540 nm. The standard used here was glucose (y = 0.7377x − 0.0061, R^2^ = 0.9976). The reducing sugar contents were calculated using the following formula:

(4) $$Reducing\;sugar\;content\left(\%\right)=\frac{C\times V_{t}}{m\times V_{s}}\times 100$$

where *C* is the amount of sugar calculated from the standard curve (mg), *V_t_* is the volume of the extracts (mL), *m* is the weight of the sample (mg), and *V_s_* is the volume of reducing sugar test solution (mL).

### 3.9. Titratable Acidity Content

Titratable acidity in mulberry fruit was determined by Chinese Standard GB 12293-1990 [36]. Briefly, each mulberry fruit was accurately weighed as 5.0 g and mixed with 5 mL of neutral distilled water. The mixture was homogenized, and then the volume was set to 25 mL with neutral distilled water. After that, the mixture was heated in a 70 °C water bath for 30 min and cooled to room temperature. The supernatant was titrated with 0.1 mol/L NaOH solution to pH 8.1, and the volume of NaOH solution used for titration was recorded. The titratable acidity of the sample was expressed as the percentage of the citric acid content, calculated according to the following formula:

(5) $$Titratable\;\mathrm{acidity}\;\left(\%\right)=\frac{c\times V_{1}\times k}{V_{0}}\times \frac{25}{m}\times 100$$

where *c* is the concentration of NaOH standard solution (0.1 mol/L), *V_1_* is the volume consumed of NaOH standard solution when titrated (mL), *V_0_* is the volume of sample solution for titration (mL), 25 is the constant volume of sample leaching (mL), *m* is the quality of samples (g), and *k* is a conversion factor for crystalline citric acid (0.070).

### 3.10. Antioxidant Activity

#### 3.10.1. Total Antioxidant Capacity-FRAP Assay

An antioxidant capacity assay kit (FRAP method) was used to evaluate the total antioxidant activity of the mulberry fruit extracts. The FRAP working solution consisted of tripyridyltriazine (TPTZ) solution, a detection buffer, and TPTZ dilution solution (1:1:10, *v*/*v*). After being incubated at 37 °C, the FRAP working solution should be used within 2 h. The working solution (180 μL) was mixed with 5 μL of mulberry fruit extracts and the blank (70% aqueous ethanol), and then the mixture was incubated at 37 °C for 5 min and the absorbance was measured at 593 nm. A total of 5 μL of five different concentrations of FeSO_4_ (0.1, 0.2, 0.5, 1 and 2 mmol Fe^2+^/L) also mixed with working solution (180 μL) and the absorbance was determined as mentioned above to obtain the standard curve. The antioxidant activity of samples was expressed as mM FeSO_4_ equivalents.

#### 3.10.2. DPPH Radical-Scavenging Capacity

The DPPH radical-scavenging capacity of the mulberry fruit extracts was evaluated by a DPPH assay with slight modifications [37]. Briefly, 100 μL of 0.1 mmol/L DPPH ethanol solution was added to 100 μL of each fruit extract. The mixture was well mixed and then placed in the dark environment to incubate for 30 min. Finally, the absorbance was measured at 517 nm. The DPPH radical-scavenging effect was expressed as the percentage inhibition calculated using the following equation:

(6) $$DPPH\;radical-scavenging\;effect\;\left(\%\right)=\frac{A_{a}-A_{b}}{A_{a}}\times 100\%$$

where *A_a_* and *A_b_* represent the blank and sample absorbance, respectively.

#### 3.10.3. Hydroxyl Radical Scavenging Activity Assay

The hydroxyl radical-scavenging capacity of the mulberry fruit extracts was evaluated using Wu et al.’s method [38], with slight modifications. Briefly, 1 mL of 1.5 mM FeSO_4_ and 0.3 mL of 20 mM salicylic acid was mixed with a 1 mL mulberry fruit extract. Then, 0.7 mL of 6 mM H_2_O_2_ was added to the mixture solution. Afterward, the solution was kept in a 37 °C water bath for 1 h. The ability of the mulberry fruit extracts to scavenge hydroxyl radicals was calculated using the following formula:

(7) $$Hydroxyl\;radical-scavenging\;activity\;\left(\%\right)=1-\frac{A_{1}-A_{2}}{A_{0}}\times 100\%$$

where *A*_0_ is the absorbance of the blank (70% ethanol instead samples), *A*_1_ is the test sample absorbance, and *A*_2_ is the control (deionized water instead of salicylic acid solution).

### 3.11. Statistical Analysis

All experimental results were expressed as mean ± standard deviation (SD). ANOVA was performed on the data of active ingredient, TPC, TFC, MAC, antioxidant activity, vitamin C, titratable acidity, and reducing sugar. The differences between samples in different regions were analyzed by a Duncan’s test (*p* < 0.05) using SPSS 19.0 software from IBM (USA). The PCA was also established using SPSS 19.0.

## 4. Conclusions

In this research, a total of 25 samples of different mulberry cultivars from six Chinese provinces were investigated. Through determining of the bioactive compounds and antioxidant activities, we found that ZS from Dechang had the highest active compound content and antioxidant activity relative to others cultivars. ZS from Zhenjiang and Dechang had a high C3G content (4.367 ± 0.243 mg/g and 4.055 ± 0.100 mg/g), and JSS from Dechang had a high C3R content (1.649 ± 0.018 mg/g). These two cultivars could be viewed as natural sources of C3G and C3R. Among the eight locations in the six provinces, mulberry fruits from Dechang had a higher active ingredient content and antioxidant activity than any other region, which suggests that Dechang is the most suitable place to plant mulberry fruits destined for use in health products. The present study provides meaningful information on the planting and application of mulberry fruits.

## Figures and Tables

**Table 1:** An abbreviated list of variety names of mulberry fruits.

Varieties	Abbreviation
Dashi	DS
Zhongsang 5801	ZS
Jushensang	JSS
Huayang	HY
Miaoli 1	ML
Tongshan 3	TS
Yunsang 2	YS
831A	831A
Jialing 30	JL
Hongguo 1	HG
Zijin 6	ZJ
Aoyv	AY
Qiuyv	QY

**Table 2:** The contents of total phenolic, total flavonoid, main anthocyanin, VC, reducing sugar, and titratable acidity of mulberry fruits among varieties from different areas in China.

No.	Origins	Varieties	TPC (mg GAE/g)	TFC (mg RE/g)	MAC (mg/g)	VC (mg/100 g)	Reducing Sugar	Titratable Acidity
							(%)	(%)
1	Liyang	DS	2.393 ± 0.048 ^f,g^	0.934 ± 0.054 ^k,l^	3.061 ± 0.022 ^n^	11.696 ± 0.799 ^k^	4.242 ± 0.089 ^j^	2.494 ± 0.292 ^f^
2	Liyang	ZS	1.691 ± 0.212 ^h,i^	0.335 ± 0.051 ^m^	2.481 ± 0.059 ^o^	12.532 ± 0.378 ^k^	5.157 ± 0.036 ^h^	1.661 ± 0.379 ^j,k^
3	Jurong	JSS	2.329 ± 0.178 ^f,g^	1.132 ± 0.097 ^j,k^	3.285 ± 0.030 ^j,k^	30.589 ± 0.349 ^g,h^	2.960 ± 0.059 ^n^	2.291 ± 0.088 ^f,g^
4	Jurong	DS	2.724 ± 0.079 ^j^	1.427 ± 0.142 ^h,i^	3.237 ± 0.025 ^k,l^	29.552 ± 0.745 ^h^	7.041 ± 0.053 ^e^	2.563 ± 0.140 ^f^
5	Jurong	ZS	2.326 ± 0.260 ^f,g^	1.810 ± 0.152 ^f,g^	3.792 ± 0.012 ^g^	40.636 ± 1.240 ^b–d^	4.727 ± 0.183 ^i^	2.561 ± 0.284 ^f^
6	Zhenjiang	DS	2.478 ± 0.288 ^f^	0.948 ± 0.047 ^k,l^	3.334 ± 0.022 ^j^	18.539 ± 2.016 ^j^	4.661 ± 0.012 ^i^	1.951 ± 0.023 ^h^
7	Zhenjiang	ZS	2.016 ± 0.183 ^g,h^	0.769 ± 0.080 ^l^	5.565 ± 0.124 ^b^	33.062 ± 1.433 ^f,g^	2.842 ± 0.034 ^n,o^	1.791 ± 0.048 ^i^
8	Anji	DS	1.749 ± 0.109 ^h,i^	1.848 ± 0.106 ^f^	3.629 ± 0.017 ^h^	40.000 ± 1.404 ^c,d^	0.515 ± 0.027 ^t^	2.347 ± 0.122 ^f^
9	Dechang	HY	4.433 ± 0.120 ^c^	2.874 ± 0.134 ^b^	5.046 ± 0.058 ^c^	35.795 ± 2.571 ^e,f^	3.259 ± 0.018 ^m^	5.284 ± 0.379 ^b^
10	Dechang	ML	4.383 ± 0.137 ^c^	2.798 ± 0.301 ^b,c^	4.604 ± 0.019 ^e^	32.897 ± 1.721 ^f,g^	1.323 ± 0.123 ^q^	3.946 ± 0.664 ^d^
11	Dechang	TS	2.620 ± 0.115 ^f^	2.450 ± 0.243 ^d,e^	0.966 ± 0.009 ^q^	45.712 ± 2.775 ^a^	2.557 ± 0.122 ^p^	3.467 ± 0.191 ^e^
12	Dechang	ZS	6.307 ± 0.541 ^a^	3.662 ± 0.269 ^a^	5.737 ± 0.052 ^a^	38.162 ± 1.889 ^d,e^	6.718 ± 0.148 ^f^	1.903 ± 0.145 ^h^
13	Dechang	YS	4.406 ± 0.116 ^c^	2.585 ± 0.145 ^c,d^	4.884 ± 0.027 ^d^	43.498 ± 2.124 ^a,b^	9.346 ± 0.088 ^b^	1.702 ± 0.121 ^i^
14	Dechang	831A	3.341 ± 0.194 ^d,e^	2.631 ± 0.252 ^b–d^	4.081 ± 0.043 ^f^	35.465 ± 1.838 ^e,f^	16.961 ± 0.037 ^a^	0.997 ± 0.050 ^l^
15	Dechang	JSS	2.362 ± 0.176 ^f,g^	1.862 ± 0.098 ^f^	5.657 ± 0.002 ^a^	25.830 ± 1.749 ^i^	7.594 ± 0.085 ^d^	3.362 ± 0.181 ^e^
16	Dechang	JL	1.430 ± 0.115 ^i^	1.950 ± 0.191 ^f^	3.306 ± 0.018 ^j,k^	29.093 ± 3.439 ^h^	8.527 ± 0.305 ^c^	0.397 ± 0.023 ^m^
17	Chongqing	HG	4.526 ± 0.280 ^c^	2.261 ± 0.037 ^e^	2.477 ± 0.004 ^o^	3.310 ± 0.736 ^l^	2.770 ± 0.023 ^o^	5.498 ± 0.286 ^b^
18	Chongqing	JL	2.233 ± 0.042 ^f,g^	0.719 ± 0.055 ^l^	3.156 ± 0.002 ^l,m^	38.928 ± 2.832 ^c,d^	5.546 ± 0.158 ^g^	2.552 ± 0.104 ^f^
19	Chongqing	ZS	3.551 ± 0.268 ^d^	0.840 ± 0.082 ^l^	3.627 ± 0.023 ^h^	31.213 ± 2.447 ^g,h^	4.136 ± 0.092 ^j,k^	2.271 ± 0.039 ^f,g^
20	Chongqing	YS	3.504 ± 0.089 ^d^	1.318 ± 0.166 ^i,j^	1.138 ± 0.008 ^p^	31.119 ± 0.711 ^g,h^	1.093 ± 0.038 ^r^	5.174 ± 0.525 ^b,c^
21	Chongqing	DS	4.929 ± 0.341 ^b^	1.593 ± 0.065 ^g,h^	3.186 ± 0.097 ^l,m^	28.209 ± 1.552 ^h,i^	5.569 ± 0.071 ^g^	4.762 ± 0.174 ^c^
22	Chongqing	ZJ	3.042 ± 0.236 ^e^	0.676 ± 0.107 ^l^	2.537 ± 0.026 ^o^	45.253 ± 2.611 ^a^	1.006 ± 0.031 ^r,s^	7.479 ± 0.495 ^a^
23	Chongqing	AY	2.487 ± 0.121 ^f^	0.746 ± 0.074 ^l^	3.126 ± 0.015 ^m,n^	42.155 ± 0.280 ^b,c^	3.909 ± 0.085 ^l^	7.327 ± 0.122 ^a^
24	Fengcheng	HG	3.083 ± 0.054 ^e^	1.463 ± 0.155 ^h,i^	3.523 ± 0.168 ^i^	40.506 ± 0.566 ^b–d^	0.884 ± 0.032 ^s^	1.628 ± 0.069 ^j,k^
25	Shuangcheng	QY	0.727 ± 0.101 ^j^	0.199 ± 0.023 ^m^	0.882 ± 0.006 ^q^	33.781 ± 0.896 ^f,g^	3.972 ± 0.015 ^k,l^	1.020 ± 0.126 ^l^

^a–t^ mean significantly different (*p* < 0.05) in Duncan test.

**Table 3:** The content of main anthocyanins in mulberry fruits among varieties from different areas in China.

No.	Origins	Varieties	Cyanidin-3-*O*-Glucoside (mg/g)	Pelargonidin-3-*O*-Rutinoside (mg/g)	Cyanidin-3-*O*-Rutinoside (mg/g)
1	Liyang	DS	2.056 ± 0.041 ^k^	1.005 ± 0.050 ^f,g^	-
2	Liyang	ZS	1.791 ± 0.067 ^l^	0.691 ± 0.007 ^i,j^	-
3	Jurong	JSS	2.222 ± 0.069 ^i–k^	1.062 ± 0.044 ^e–g^	-
4	Jurong	DS	2.276 ± 0.032 ^i,j^	0.961 ± 0.009 ^g^	-
5	Jurong	ZS	2.612 ± 0.015 ^f,g^	1.180 ± 0.003 ^b–d^	-
6	Zhenjiang	DS	2.094 ± 0.037 ^k^	1.240 ± 0.022 ^b^	-
7	Zhenjiang	ZS	4.367 ± 0.243 ^a^	1.198 ± 0.134 ^b,c^	-
8	Anji	DS	2.545 ± 0.092 ^f–h^	1.083 ± 0.075 ^d–f^	-
9	Dechang	HY	3.885 ± 0.061 ^c^	1.161 ± 0.003 ^c–e^	-
10	Dechang	ML	3.524 ± 0.044 ^d^	1.080 ± 0.025 ^d–f^	-
11	Dechang	TS	0.966 ± 0.009 ^m^	-	-
12	Dechang	ZS	4.055 ± 0.100 ^b^	1.270 ± 0.031 ^b^	0.412 ± 0.018 ^a^
13	Dechang	YS	3.444 ± 0.174 ^d^	1.007 ± 0.152 ^f,g^	0.432 ± 0.038 ^a^
14	Dechang	831A	2.914 ± 0.053 ^e^	0.763 ± 0.035 ^h,i^	0.404 ± 0.057 ^a^
15	Dechang	JSS	4.008 ± 0.014 ^b,c^	1.649 ± 0.013 ^a^	-
16	Dechang	JL	2.375 ± 0.028 ^h,i^	0.612 ± 0.014 ^j,k^	0.319 ± 0.014 ^a^
17	Chongqing	HG	2.121 ± 0.011 ^j,k^	0.356 ± 0.007 ^l^	-
18	Chongqing	JL	2.581 ± 0.031 ^f,g^	0.576 ± 0.033 ^k^	-
19	Chongqing	ZS	3.017 ± 0.060 ^e^	0.610 ± 0.036 ^j,k^	-
20	Chongqing	YS	0.871 ± 0.014 ^m^	0.268 ± 0.006 ^l,m^	-
21	Chongqing	DS	2.463 ± 0.132 ^g,h^	0.723 ± 0.036 ^h,i^	-
22	Chongqing	ZJ	1.854 ± 0.005 ^l^	0.683 ± 0.031 ^i,j^	-
23	Chongqing	AY	2.509 ± 0.003 ^g,h^	0.617 ± 0.018 ^j,k^	-
24	Fengcheng	HG	2.708 ± 0.293 ^f^	0.815 ± 0.134 ^h^	-
25	Shuangcheng	QY	0.656 ± 0.006 ^n^	0.226 ± 0.007 ^m^	-

^a–^^n^ mean significantly different (*p* < 0.05) in Duncan test.

**Table 4:** The correlation coefficients of the contents of total phenolic, total flavonoid, main anthocyanin, vitamin C, reducing sugar, titratable acidity, antioxidant capacity (FRAP, DPPH, hydroxyl radical scavenging capacity) and the main anthocyanin (cyanidin-3-*O*-glucoside, cyanidin-3-*O*-rutinoside).

	TPC	TFC	MAC	VC *	RS	TA	FRAP	DPPH	OH Scavenging	C3G	C3R
TPC	1.000	0.659	0.376	0.048	0.030	0.315	0.686	0.483	−0.117	0.415	0.117
TFC	0.659	1.000	0.473	0.193	0.286	−0.025	0.807	0.183	−0.411	0.463	0.251
MAC	0.376	0.473	1.000	0.110	0.301	−0.171	0.656	0.116	−0.141	0.981	0.852
VC	0.048	0.193	0.110	1.000	−0.028	0.083	0.124	0.014	0.152	0.133	−0.038
RS	0.030	0.286	0.301	−0.028	1.000	−0.433	0.248	0.056	−0.198	0.244	0.151
TA	0.315	−0.025	−0.171	0.083	−0.433	1.000	0.037	0.398	0.556	−0.102	−0.180
FRAP	0.686	0.807	0.656	0.124	0.248	0.037	1.000	0.228	−0.370	0.610	0.541
DPPH	0.483	0.183	0.116	0.014	0.056	0.398	0.228	1.000	0.349	0.144	−0.014
OH scavenging	−0.117	−0.411	−0.141	0.152	−0.198	0.556	−0.370	0.349	1.000	−0.114	−0.072
C3G	0.415	0.463	0.981	0.133	0.244	−0.102	0.610	0.144	−0.114	1.000	0.758
C3R	0.117	0.251	0.852	−0.038	0.151	−0.180	0.541	−0.014	−0.072	0.758	1.000

VC *, vitamin C; RS, reducing sugar; TA, titratable acidity; OH scavenging, hydroxyl radical scavenging capacity; C3G, cyanidin-3-*O*-glucoside; C3R cyanidin-3-*O*-rutinoside.

## Data Availability

Not applicable.

## Sample Availability

Not available.
